# Increased *Slc12a1* expression in β-cells and improved glucose disposal in *Slc12a2* heterozygous mice

**DOI:** 10.1530/JOE-15-0327

**Published:** 2015-12

**Authors:** Saeed Alshahrani, Mohammed Mashari Almutairi, Shams Kursan, Eduardo Dias-Junior, Mohamed Mahmoud Almiahuob, Lydia Aguilar-Bryan, Mauricio Di Fulvio

**Affiliations:** 1 Department of Pharmacology and Toxicology, Boonshoft School of Medicine, Wright State University, 3640 Colonel Glenn Highway, 216 HSB, Dayton, Ohio, 45435, USA; 2 Pacific Northwest Diabetes Research Institute, Seattle, Washington, 98122, USA

**Keywords:** NKCC1, NKCC2, β-cell, glucose homeostasis, insulin secretion

## Abstract

The products of the *Slc12a1* and *Slc12a2* genes, commonly known as Na^+^-dependent K^+^2Cl^−^ co-transporters NKCC2 and NKCC1, respectively, are the targets for the diuretic bumetanide. NKCCs are implicated in the regulation of intracellular chloride concentration ([Cl^−^]_i_) in pancreatic β-cells, and as such, they may play a role in glucose-stimulated plasma membrane depolarization and insulin secretion. Unexpectedly, permanent elimination of NKCC1 does not preclude insulin secretion, an event potentially linked to the homeostatic regulation of additional Cl^−^ transporters expressed in β-cells. In this report we provide evidence for such a mechanism. Mice lacking a single allele of *Slc12a2* exhibit lower fasting glycemia, increased acute insulin response (AIR) and lower blood glucose levels 15–30 min after a glucose load when compared to mice harboring both alleles of the gene. Furthermore, heterozygous expression or complete absence of *Slc12a2* associates with increased NKCC2 protein expression in rodent pancreatic β-cells. This has been confirmed by using chronic pharmacological down-regulation of NKCC1 with bumetanide in the mouse MIN6 β-cell line or permanent molecular silencing of NKCC1 in COS7 cells, which results in increased NKCC2 expression. Furthermore, MIN6 cells chronically pretreated with bumetanide exhibit increased initial rates of Cl^−^ uptake while preserving glucose-stimulated insulin secretion. Together, our results suggest that NKCCs are involved in insulin secretion and that a single *Slc12a2* allele may protect β-cells from failure due to increased homeostatic expression of *Slc12a1*.

## Introduction

Chronic hyperglycemia due to β-cell dysfunction and reduced tissue sensitivity to insulin are the main features of type-2 diabetes mellitus (DeFronzo 2004). Under normal circumstances, glucose promotes insulin secretion in a biphasic way. The first phase is represented by an acute and sharp insulin response from granules, pre-docked at the plasma membrane, followed by a more sustained and slower second step of hormone release, from granules subsequently recruited from reserved pools, which persists until euglycemia is attained (Henquin *et al*. 2002). The initial triggering signals provoked by glucose are well understood and involve a series of steps frequently reduced to the so-called consensus model. These events are: facilitated diffusion of glucose into β-cells, phosphorylation of the sugar and consumption via glycolysis, increased ATP/ADP ratio, closure of ATP-sensitive K^+^ (K_ATP_)-channels, plasma membrane depolarization, increased Ca^2+^ influx due to opening of voltage-dependent Ca^2+^ channels and exocytosis of insulin stored in primed granules (Henquin 2000).

K_ATP_-channels play a crucial role in the transduction of metabolic signals into electrical responses, regulating insulin release. Inactivating or activating mutations in one of the two subunits forming this channel, i.e., the sulfonylurea receptor-1 gene (SUR1, *ABCC8*) or the inward rectifier K^+^ channel (Kir_6.2_, *KCNJ11*), cause congenital hyperinsulinemic hypoglycemia (Thomas *et al*. 1995) and diabetes in neonates and infants (Babenko *et al*. 2006). However, mice lacking *Abcc8* or *Kcnj11* exhibit mildly impaired glucose homeostasis (Miki *et al*. 1998, Seghers *et al*. 2000, Szollosi *et al*. 2007, Rosario *et al*. 2008) due to a conserved amplifying pathway of insulin secretion (Nenquin *et al*. 2004). Rodent β-cells exhibit anionic depolarizing currents in response to glucose that are independent of functional K_ATP_-channels (Best *et al*. 2010) with one related to Cl^−^ ions (Di Fulvio *et al*. 2014). Thermodynamically, the depolarizing efflux of Cl^−^ from β-cells is possible because [Cl^−^]_i_ is kept higher than the Nernstian equilibrium (Sehlin 1978, Eberhardson *et al*. 2000, Best 2005). Accordingly, Cl^−^ is inwardly transported into β-cells against its electrochemical gradient mainly by the bumetanide (BTD)-sensitive Na^+^K^+^2Cl^−^ co-transporter-1 (NKCC1) (Majid *et al*. 2001, Best 2005). This has been demonstrated in dispersed rodent β-cells (Sandstrom & Sehlin 1988, Sandstrom 1990, Best 2005) and β-cell lines (Alshahrani & Di Fulvio 2012). Expectedly, acute inhibition of Cl^−^ channels, which dissipate the outwardly directed Cl^−^ gradient set forth by Cl^−^ loaders, also alters β-cell plasma membrane depolarization and insulin secretion. These Cl^−^ channels include the volume regulated (Best *et al*. 2010), cystic fibrosis conductance regulator (CFTR) (Edlund *et al*. 2014, Guo *et al*. 2014), Ca^2+^-activated (Kozak & Logothetis 1997, Edlund *et al*. 2014), GABA-gated (Braun *et al*. 2010) and others of unknown molecular identity (Kinard & Satin 1995, Kinard *et al*. 2001). Consequently, three key concepts emerge from these results: β-cells keep [Cl^−^]_i_ above predicted thermodynamic equilibrium, Cl^−^ loaders functionally predominate over extruders and the maintenance and regulation of [Cl^−^]_i_ in β-cells modulate insulin secretion (Di Fulvio *et al*. 2014).

Overall, β-cells' long-term adaptive responses include activation of key proteins involved in the stimulus-secretion coupling and/or regulation of gene programs necessary for β-cells to keep up with the vast physiological and pathological demands (Cerf 2013, Oh 2015). Several mouse models exemplified the concept that the expression of a gene/group of genes in response to deleting one may be restricted to specific cell types or translate into unique overall responses when compared to the WT (Kafri *et al*. 2006). For instance, mice lacking *Slc12a2* genes (NKCC1^KO^) do not exhibit a hyperglycemic/diabetic phenotype (Alshahrani & Di Fulvio 2012), an unexpected outcome based on the observation that diuretics acutely impair insulin secretion in rodents (Di Fulvio *et al*. 2014) and alter insulin secretion in humans (Jackson 2006). Also, NKCC1^KO^ mice exhibit paradoxically high insulin responses and rapid glucose clearance (Alshahrani & Di Fulvio 2012). Although the nature of possible mechanisms for these observations in the absence of *Slc12a2* in β-cells remains unknown, current evidence from rodent secretory epithelia or neurons suggests that different genes encoding anion exchangers or Cl^−^ channels are activated in the absence of NKCC1 (Grubb *et al*. 2000, 2001, Walker *et al*. 2002, Wall *et al*. 2006, Schobel *et al*. 2012, Haering *et al*. 2015, Taylor-Burds *et al*. 2015). To the best of our knowledge there is no information for humans.

β-cells are endowed with several alternative mechanisms to regulate [Cl^−^]_i_ including Cl^−^ co-transporters, exchangers and channels (Di Fulvio *et al*. 2014). The BTD-sensitive Cl^−^ loader NKCC2 has been found in rodent islets as well as rat (INS-1E, RIN-5mF) and mouse (MIN6) β-cell lines (Bensellam *et al*. 2009, Alshahrani & Di Fulvio 2012, Alshahrani *et al*. 2012). Interestingly, NKCC1^KO^ islets express more NKCC2 relative to NKCC1^WT^ islets while heterozygous mice exhibit intermediate levels (Alshahrani *et al*. 2012), mimicking the observation when using chronic high glucose, which decreases NKCC1 mRNA while increasing that of NKCC2 (Bensellam *et al*. 2009). Given the functional interplay between *Slc12a1* and *Slc12a2* genes in β-cells, the present work tested the hypothesis that phenotypically normal mice lacking a single NKCC1 allele (NKCC1^HE^) are glucose tolerant due to increased *Slc12a1* gene expression. Validating the hypothesis, NKCC1^HE^ mice have enhanced acute insulin response (AIR) and increased initial glucose disposal. Furthermore, NKCC2 expression responds to NKCC1 inhibition in β-cells and these cells show increased rates of Cl^−^ accumulation and a normal secretory response, generally supporting the existence of a functional relationship between *Slc12a1* and *Slc12a2* genes aimed at modulating [Cl^−^]_i_ in β-cells to preserve the secretory response.

## Material and methods

### Materials

This study used human recombinant insulin from Eli Lilly & Co, protease/phosphatase inhibitors, pre-casted Tris-HEPES 4–20% SDS–PAGE gels, West Pico 34080 chemiluminescence kit and anti-human NKCC1 chicken IgY (ckNKCC1) from Pierce (Thermo Scientific, Rockford, IL, USA). BTD, glucose and general reagents were from Sigma. Human NKCC2 goat IgG from Everest Biotech (Oxfordshire, UK), β-actin IgM from Developmental Studies Hybridoma Bank (Iowa City, IA, USA) and anti-guinea pig insulin antibodies were from Cell Marque (Rocklin, CA, USA). Conjugated secondary antibodies were from Jackson ImmunoResearch (West Grove, PA, USA).

### Animals

The Institutional Animal Care and Use Committee approved all experiments with animals. Male and female mice 4 weeks old NKCC1^HE^ and NKCC1^WT^ were used. Weight was 10.7±0.7 and 11.2±0.5 g respectively (*n*=14–32, *P*=0.466); both were provided comparable food and water intake and allowed to feed *ad libitum* and were housed under 12 h light:12 h darkness cycles. The most relevant phenotype and secretory response of the NKCC1^KO^ mice have been published (Flagella *et al*. 1999, Alshahrani & Di Fulvio 2012).

### Islet isolation and cell culture

Primary islets and low passage MIN6 and COS7 cells were cultured as described (Miyazaki *et al*. 1990, Alshahrani & Di Fulvio 2012). Of note, COS7 cells express abundant NKCC1 (Singh *et al*. 2015) and very low levels of NKCC2. COS7 cells were stably transfected as indicated (Singh *et al*. 2015).

### Intra-peritoneal glucose and insulin tolerance tests

We used the International Mouse Phenotyping Resource of Standardized Screens and published recommendations to evaluate glucose homeostasis (Ayala *et al*. 2010). Glucose and insulin tolerance tests (GTT and ITT respectively) were performed by i.p. administration of 2 g/kg d-glucose or 0.75 U/kg of insulin respectively. Blood glucose was determined by using a calibrated glucometer (FreeStyle-Lite, Abbott Park, IL, USA). The effect of 50 mg/kg BTD on GTT was tested 5 min after the i.p. injection.

### Immunoblotting

Protein were extracted from ∼90% confluent MIN6 or COS7 cells using a lysis buffer plus protease/phosphatase inhibitors. Proteins (50–100 μg) were run on pre-casted gels, electro-transferred and blotted as described (Singh *et al*. 2015). Antibodies against NKCC1, NKCC2 or β-actin were used at 1:1000, 1:250 and 1:1000 dilutions respectively. Appropriate secondary antibodies were employed to develop blots by chemiluminescence, using the BioRad image analyzer (Chemi-Doc MP Imaging system, Hercules, CA, USA). Densitometric analysis was performed as described (Singh *et al*. 2015).

### Immunofluorescence microscopy

Tissues were obtained from perfused mice, placed in 4% *p*-formaldehyde (PFA) overnight at 4 °C, dehydrated in 20% sucrose-4% PFA and paraffin embedded. Blocks were sectioned at 5 μm (AML Laboratories, Inc., Baltimore, MD, USA), post-fixed as above and deparaffinized in xylene. Antigens were retrieved in sodium citrate buffer (10 mM) at 100 °C for 30 min, blocked and incubated with anti-NKCC1, -NKCC2 or -insulin antibodies (1:500, 1:100 or 1:500 respectively). Sections were washed and incubated with AF488-, Cy3- or DyLight-conjugated secondary antibodies 2 h at room temperature in darkness. When appropriate, 4′,6-diamidino-2-phenylindole (DAPI)-containing mounting media was used to counterstain cell nuclei. Digital images were taken by using the BX51 system fluorescence microscope (Olympus Corp., Tokyo, Japan) connected to a Spot 5.1 digital camera (SPOT Imaging Solutions, Diagnostic Instruments, Inc., Sterling Heights, MI, USA) coupled to MetaVue software (Molecular Devices, Sunnyvale, CA, USA). COS7 were immunolabeled as described (Singh *et al*. 2015).

### Determination of cellular Cl^−^

The total intracellular content of Cl^−^ in MIN6 cells was determined by using calibrated ion-selective electrodes (Orion-Thermo Scientific) as described (Northrop 1948, Sanderson 1952, Weinstein & Jennings 1959) with modifications (Singh *et al*. 2015). Briefly, cells were seeded onto six-well plates and grown to ∼80% confluence. Cells were washed and depleted of endogenous Cl^−^ by pre-incubating them 1 h at room temperature in an isotonic (ISO, ∼300 mOsm/kg) solution free of Cl^−^ ions (in mM: 0.83 Na_2_HPO_4_, 1 Mg_2_SO_4_, 20 HEPES, 10 mannose and 130, 5 and 2 gluconate salts of Na^+^, K^+^ and Ca^2+^, respectively). To assess Cl^−^ uptake, cells were allowed to recover Cl^−^ in ISO containing physiological Cl^−^ for 5 min. For kinetic analysis, Cl^−^ uptake was terminated at 0, 5, 10, 30 or 60 min by placing cells on ice and washing them in ice-cold Cl^−^-free ISO. The total cellular Cl^−^ content was released in 0.25 M NaOH, neutralized with glacial acetic acid and measured. Net Cl^−^ uptake in cells was calculated and expressed as nanomole/liter of Cl^−^ per microgram of total protein. BTD-sensitive Cl^−^ uptake was defined as the difference between Cl^−^ accumulated to that obtained in the presence of BTD 5 min. The long-term effect of BTD on Cl^−^ accumulation was assayed after 16 h pre-incubation. Cells were then depleted of endogenous Cl^−^ in Cl^−^-free ISO plus BTD, as indicated, and allowed to re-accumulate the anion 5 min in ISO with or without BTD.

### Insulin determination

Insulin concentration in plasma or cell/islet media was determined by using a mouse ultrasensitive immunoassay (Alpco, Salem, NH, USA). Blood samples were obtained from 12 h fasted mice (basal) and/or after a single i.p. injection of glucose (2 g/kg). Blood (∼50 μl) was collected with the help of heparinized capillary tubes (Scientific Glass, Inc., Rockwood, TN, USA) and placed in thin-walled PCR tubes on ice to collect plasma. Plasma was stored at −80 °C until use. Insulin secretion is expressed as percentage change relative to basal values i.e., in response to 5.5 mM glucose. Results are normalized to total insulin content.

### Statistical analysis

Data are expressed as means±s.e.m. The difference between means of two populations was determined using Student's two-tailed *t*-test after a preliminary F-test to determine homogeneity of within-group variances. The differences and significances in blood glucose or plasma insulin between more than two groups were determined using one-way ANOVA (GraphPad Prism Software, San Diego, CA, USA). The trapezoidal method was used to calculate the area under the curve (AUC) of GTT and ITT. The homeostatic model assessment (HOMA) was calculated from fasting plasma insulin (pmol/l) and blood glucose (mM): HOMA=(Insulin)×(Glucose)/135. Statistical significance was considered when *P*<0.05.

## Results

### NKCC1^HE^ mice exhibit increased glucose disposal

**Figure 1:**
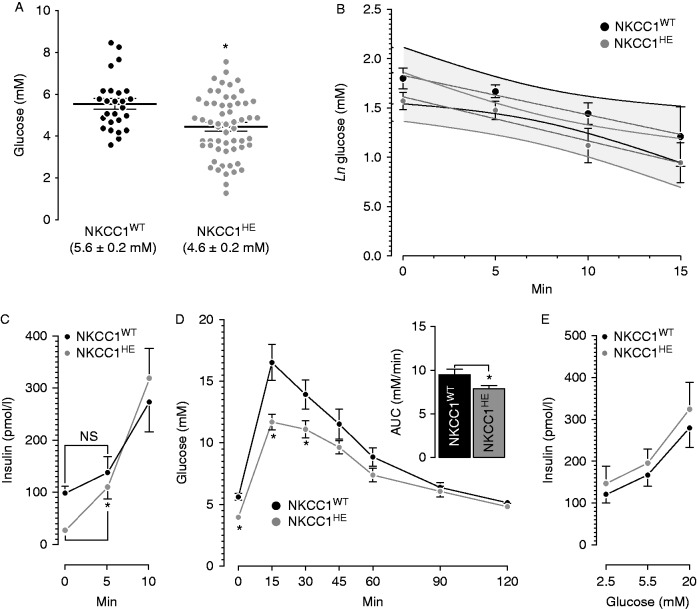
Basal glycemia, glucose disposal, plasma insulin and insulin responses *in vitro* and *in vivo* in NKCC1^HE^ mice. (A) Blood glucose levels in fasted NKCC1^WT^ (black dots, *n*=26) and NKCC1^HE^ mice (grey dots, *n*=58). Values are stated for each genotype and expressed as the means±s.e.m. (*P=0.003). (B) Insulin-induced hypoglycemia in fasted NKCC1^WT^ (black dots, *n*=5) and NKCC1^HE^ mice (grey dots, *n*=6). Results are plotted as natural logarithms of initial blood glucose, i.e., before insulin injection vs time in minutes. The differences between the slopes of each curve (grey lines), calculated by linear regression analysis, were not statistically different (*P*=0.816). The 95% confidence limits are indicated as overlapped shaded areas. (C) Basal and glucose-stimulated plasma insulin in fasted NKCC1^WT^ (black dots, *n*=3–7) and NKCC1^HE^ mice (grey dots, *n*=7–12). Plasma insulin levels either basal or 5 min post-glucose stimuli were not different (NS) in NKCC1^WT^ (basal: 107.1±5.2 pmol/l, 5 min: 137.7±30.9 pmol/l, *P*=0.443) but were in NKCC1^HE^ mice (basal: 25.8±2.4 pmol/l, 5 min: 109.8±21.9 pmol/l, *P=0.010). (D) Intra-peritoneal glucose tolerance tests in NKCC1^WT^ (black dots, *n*=7) and NKCC1^HE^ mice (grey dots, *n*=10). Basal blood glucose in NKCC1^WT^ and NKCC1^HE^ mice is 5.6±0.3 and 4.0±0.3 mM respectively (*P=0.001). Glycemia 15 and 30 min post-glucose stimulus was significantly lower in NKCC1^HE^ mice relative to NKCC1^WT^ (*P<0.05). Inset: areas under each GTT curve (NKCC1^WT^=9.5±0.6 mM/min, NKCC1^HE^=7.9±0.4, *P=0.027). (E) Insulin secretion from equivalent numbers of NKCC1^WT^ and NKCC1^HE^ islets (black and grey dots, respectively) challenged with the indicated glucose concentrations. Results are expressed as mean insulin (pmol/l)±s.e.m. present in the culture media (*n*=5).

NKCC1^WT^ and NKCC1^HE^ mice exhibit comparable random blood glucose (8.7±0.3 and 8.3±0.8 mM respectively, *n*=8–12, *P*=0.943). However, as shown in Fig. 1A, fasting blood glucose is significantly low in NKCC1^HE^ mice relative to NKCC1^WT^ (*P=0.003) matching the significantly reduced basal plasma insulin in NKCC1^HE^ relative to NKCC1^WT^ mice (25.8±2.4 vs 107.1±5.2 pmol/l respectively, *P=0.017). The HOMA-IR calculated was 0.9 and 4.4 for NKCC1^HE^ and NKCC1^WT^ mice, respectively, suggesting that NKCC1^HE^ mice are more sensitive to glucose, insulin or both than NKCC1^WT^. Figure 1B shows the results for the ITTs; the rates of glucose disappearance in NKCC1^WT^ and NKCC1^HE^ after insulin challenges are not significantly different indicating that these mice's sensitivity to exogenous insulin is similar. To determine the AIR in NKCC1^HE^ mice, plasma insulin levels were determined 5 and 10 min after a single dose of glucose. As shown in Fig. 1C, NKCC1^HE^ mice exhibit significantly higher AIR relative to NKCC1^WT^ 5 min post-glucose (fold increase from basal: 4.2±0.6 (NKCC1^HE^, *P=0.020) vs 0.5±0.2 (NKCC1^WT^, *P*=0.400)) suggesting that NKCC1 haploinsufficiency influences AIR *in vivo*. To determine the significance of these results, NKCC1^HE^ mice were subjected to GTTs. Figure 1D shows that NKCC1^HE^ mice had significantly lower glycemia 15–30 min post-glucose challenge relative to NKCC1^WT^ (*P<0.05, *n*=10). Accordingly, AUC of NKCC1^HE^ mice GTT is significantly lower than that of NKCC1^WT^ (inset Fig. 1D) suggesting that NKCC1^HE^ mice are more tolerant to glucose than NKCC1^WT^. To examine the sensitivity of the secretory response *in vitro*, islets from NKCC1^WT^ and NKCC1^HE^ were challenged with non-insulinotropic or stimulatory glucose (2.5–5.5 mM and 20 mM respectively). Figure 1E shows that the insulin responses from islets of either genotype are similar (*P*>0.05), indicating that half-expression of NKCC1 does not impact islet sensitivity to glucose. In conclusion, hemi-expression of NKCC1 in mice results in ∼20% faster glucose clearance due to increased initial rates of insulin secretion *in vivo*.

### Bumetanide impairs glucose tolerance in NKCC1^HE^ mice

**Figure 2:**
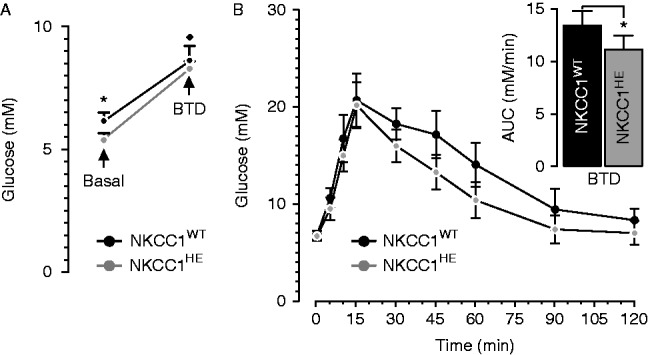
Effect of BTD on fasting blood glucose and glucose clearance in NKCC1^HE^ mice. (A) Glucose levels were determined in two groups (*n*=10 each) of fasted NKCC1^WT^ and NKCC1^HE^ mice (black and grey dots respectively) before (basal, *P=0.035) and 5 min after i.p. injection of 50 mg/kg of BTD (*P<0.05 relative to basal). (B) Intra-peritoneal glucose tolerance tests performed in fasted NKCC1^WT^ and NKCC1^HE^ mice (filled and shaded dots respectively) 5 min after BTD injection (50 mg/kg). Inset: AUC of each GTT curve (*P=0.010).

To determine the effect of BTD on glucose levels and glucose tolerance in NKCC1^HE^ mice, blood glucose was determined under basal conditions and 5 min after a single dose of the diuretic. As shown in Fig. 2A, acute BTD significantly increases fasting glycemia in both mice models. Nevertheless, blood glucose post-BTD was not different in NKCC1^WT^ and NKCC1^HE^ mice (8.5±0.8 vs 7.7±0.7 mM respectively, *P*=0.344), in spite of significantly lower basal glycemia in NKCC1^HE^ relative to NKCC1^WT^ mice (*P=0.035). Thus, to dissect this discrepancy, we determined the extent to which BTD impacts glucose clearance. To this end, NKCC1^HE^ mice were subjected to a GTT 5 min after a single i.p. dose of BTD (50 mg/kg). As shown in Fig. 2B and its inset, BTD deteriorates glucose clearance in NKCC1^WT^ when compared to untreated mice (shown in Fig. 1D). Indeed, AUC for NKCC1^WT^ treated with BTD is significantly higher than that of untreated mice (13.4±1.4 and 9.5±0.6 mM/min respectively, *P=0.021), along the same line as our previous results (Sandstrom 1988, Alshahrani & Di Fulvio 2012). Notably, BTD significantly impaired glucose tolerance in NKCC1^HE^ mice relative to control (11.1±1.3 and 7.9±0.4 mM/min respectively, *P=0.010). However, the extent to which BTD worsened glucose clearance in NKCC1^WT^ and NKCC1^HE^ mice are similar (29.1±5.7% and 28.8±2.5% respectively) indicating that basal glycemia and glucose tolerance in mice of both genotypes are equally sensitive to the diuretic and therefore independent of the *Slc12a2* genetic dose.

### NKCC2 expression in β-cells is linked to NKCC1

**Figure 3:**
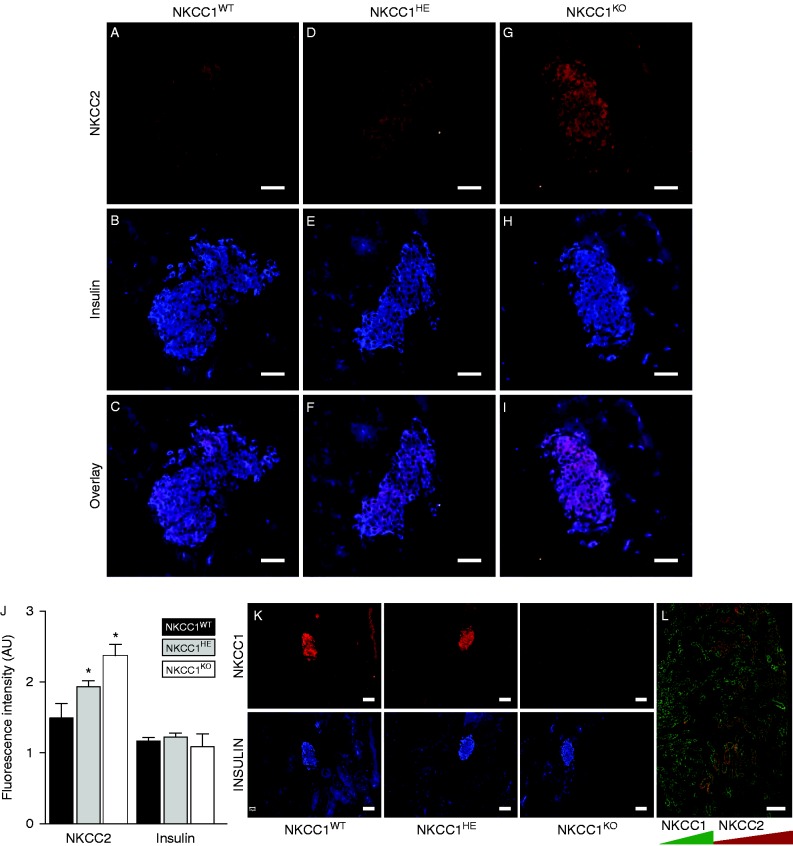
NKCC2 expression in NKCC1 mice pancreatic β-cells. (A, B, C, D, E, F, G, H and I) Representative immunofluorescence microscopy images of pancreas slides obtained from NKCC1^WT^, NKCC1^HE^ and NKCC1^KO^ mice immunolabeled against endogenous NKCC2 (A, D and G) or insulin (B, E and H), using Cy3- and DyLight-labeled secondary antibodies (red (NKCC2) and blue (insulin), respectively). Also shown are superimposed pictures of NKCC2- and insulin-labeled images to visualize co-localization of both antigens (red+blue=pink (C, F and I)). (J) Semi-quantitation of red and blue pixel number corresponding to NKCC2 and insulin, respectively. Shown are the results obtained from at least ten islets from three or more sections. Data are represented as mean fluorescence intensity in arbitrary units±s.e.m. (*P<0.05). (K) Representative images of NKCC1^WT^, NKCC1^HE^ and NKCC1^KO^ pancreas sections immunolabeled against NKCC1 or insulin and developed using fluorescently labeled secondary antibodies: Cy3 (NKCC1, red) and DyLight (insulin, blue). (L) Co-immunolocalization of NKCC1 (green) and NKCC2 (red) in kidney slides of NKCC1^WT^ mice. Shown is a representative image obtained at low magnification (20×). Scale bars represent 50 μm.

**Figure 4:**
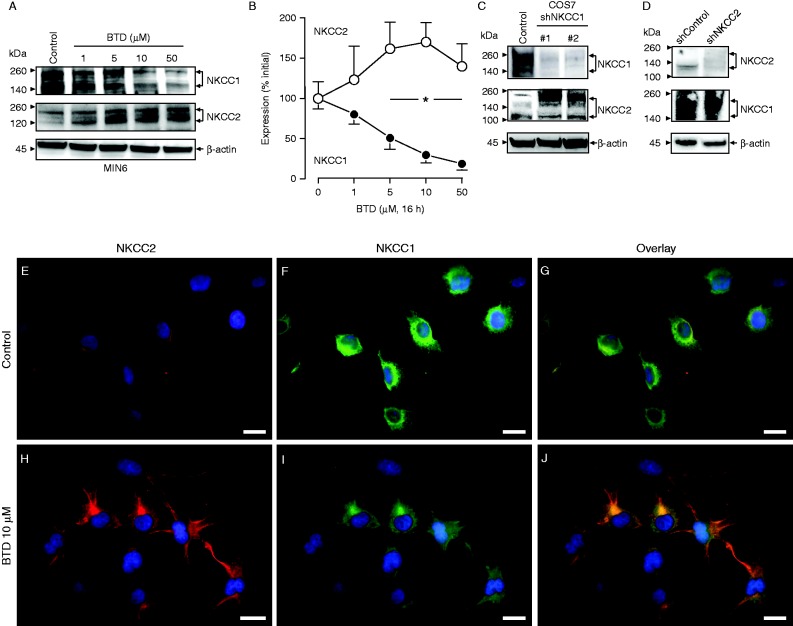
Expression relationship between NKCC1 and NKCC2 in β-cells. (A) Representative immunoblot of protein extracts obtained from MIN6 (Control and treated with the indicated concentrations BTD 16 h probed against NKCC1, NKCC2 or β-actin (internal loading control)). (B) Densitometry analysis of at least three independent experiments. Results are expressed as percentage change in densitometry intensity (arbitrary units relative to initial values (*P<0.05)). (C and D) Representative western blots of COS7 cells (Control) and stably depleted of NKCC1 (shNKCC1) (C) or COS7 Control and depleted of NKCC2 (shNKCC2) (D) probed against NKCC1 and NKCC2. Note that the NKCC2 blot in C has been overexposed to visualize NKCC2 in COS7 control. (E, F, G, H, I and J) Representative immunofluorescence microscopy images of COS7 cells cultured in the absence (Control, E, F and G) or in the presence of BTD for 16 h (BTD 10 μM, H, I and J). Co-expression of immunoreactive NKCC2 and NKCC1 was developed by using fluorescently labeled secondary antibodies: Cy3 (NKCC2, red) and AF488 (NKCC1, green). The cell nuclei were counterstained using DAPI. Scale bars represent 10 μm.

When compared against NKCC1^WT^, NKCC1^HE^ mice express ∼50% of NKCC1 transcripts in the pancreas (Flagella *et al*. 1999) and ∼30% NKCC1 protein levels in purified islets (Alshahrani *et al*. 2012). Therefore, we searched for potential homeostatic changes in NKCC2 expression in NKCC1^HE^ islets. As shown in Fig. 3A, B and C and confirming previous results (Alshahrani *et al*. 2012, Alshahrani & Di Fulvio 2012), NKCC2 immunoreactivity localizes within the pancreatic islet in NKCC1^WT^ mice, primarily in β-cells as the co-staining with insulin indicates. In comparison, NKCC2 immunoreactivity in islet β-cells of NKCC1^HE^ and NKCC1^KO^ mice (Fig. 3D, E, F, G, H and I respectively) seemed to be increased relative to NKCC1^WT^. It is important to note, as shown in Fig. 3G, that NKCC2 antibodies do not to cross-react with NKCC1. To assess these changes, the immunofluorescence signals corresponding to NKCC2 and insulin were semi-quantified *in silico*. Figure 3J shows that NKCC2 immunoreactivity localized to NKCC1^KO^ islets is significantly higher than that of NKCC1^WT^, whereas intermediate values are estimated for NKCC1^HE^ suggesting that NKCC2 expression increases when NKCC1 is reduced or absent. To support these results, pancreatic tissue sections from NKCC1 mice of the three genotypes were immunostained against NKCC1. As shown in Fig. 3K, NKCC1 expression is higher in the islet of NKCC1^WT^ when compared to NKCC1^HE^ mice, extending previous results (Alshahrani *et al*. 2012), but absent in the islet of NKCC1^KO^, thus validating the specificity of NKCC1 antibodies. To validate these observations, we tested the specificity of NKCC2 antibodies. As shown in Fig. 3L, NKCC2 antibodies detect its antigen in the outer medullary region and cortex of the kidney, whereas NKCC1 localizes to the inner medulla, the main sites for these transporters in the mammalian kidney (Gamba 2005). To support the previous observations, we used the mouse β-cell line MIN6 that had been pre-treated chronically with BTD (1–50 μM) to decrease NKCC1 protein levels and determine NKCC2 protein expression under these conditions. Preliminary results indicated that NKCC1 expression significantly decreased 4–8 h after BTD (10 μM) treatment, reaching maximal down-regulation at 16 h (not shown). As shown in Fig. 4A and B, BTD dose-dependently reduces NKCC1 expression in MIN6 reaching a valley at 10 μM. In parallel, chronic pre-treatment of MIN6 with BTD also results in increased NKCC2 protein expression (Fig. 4A). In fact, densitometry analysis of at least three independent immunoblotting experiments demonstrates that BTD decreases NKCC1 levels by 70%±9% while increasing NKCC2 by 71%±24% relative to baseline (Fig. 4B), supporting our previous hypothesis that NKCC1 down-regulation increases NKCC2 expression.

To verify and extend these results, the expression relationship between NKCC1 and NKCC2 was analyzed in COS7 cells after stable silencing of NKCC1 or NKCC2. As shown in Fig. 4C and D, silencing of NKCC1 in COS7 cells results in increased NKCC2 expression, whereas elimination of endogenous NKCC2 does not impact NKCC1 expression at all. In parallel, NKCC2 immunoreactivity was determined in normal COS7 cells treated with vehicle or BTD. As shown in Fig. 4E, F and G, under control conditions COS7 cells express NKCC2 in intracellular compartments and at very low levels relative to NKCC1 (estimated NKCC2:NKCC1 ratio=0.35±0.05 AU). On treatment with 10 μM BTD, the NKCC2/NKCC1 immunoreactivity ratio significantly increase to 1.01±0.15 AU (Fig. 4H, I and J). Notably, COS7 cells pre-incubated 16 h with 10 μM BTD show visible changes in NKCC1 and NKCC2 cellular distribution (Fig. 4H, I and J) with increased endogenous NKCC2 expression in response to BTD. Similar to the increased immunoreactivity toward the edges of the cells, NKCC1, decreased in response to the diuretic, appears retained intracellularly. These results strongly suggest that rodent β-cells with decreased NKCC1 levels also have increased NKCC2 expression.

### NKCC2 up-regulation in β-cells correlates with increased Cl^−^ uptake and insulin secretion

**Figure 5:**
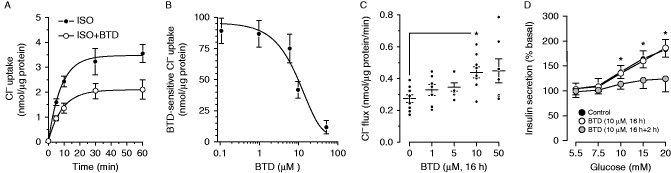
BTD-sensitive Cl^−^ uptake and insulin secretion in MIN6 cells. (A) Cl^−^ recovery in MIN6 depleted of intracellular Cl^−^ under control (ISO, filled dots) or in the presence of 10 μM BTD (ISO+BTD, open dots). Cl^−^ uptake equilibrated at basal levels after ∼15 min at room temperature and a final physiological Cl^−^ concentration of ∼140 mM. Results are expressed as mean±s.e.m. (*n*=10). The initial uptake rate is approximately linear during the first 5–10 min of the reaction. Thereafter, Cl^−^ recovery followed a mono-exponential decay. (B) Dose-response curve of Cl^−^ uptake in Cl^−^-depleted MIN6 assayed 5 min after readmission of physiological Cl^−^ in the presence of BTD (0.1–50 μM). Results are expressed as the mean±s.e.m. (*n*=5). (C) Long-term effect of BTD on the initial rates of Cl^−^ uptake in MIN6. Cells were pre-incubated 16 h with the indicated concentrations of BTD and then depleted of Cl^−^ by incubating them in Cl^−^-free medium plus BTD for 1 h. Then, total Cl^−^ content was determined 5 min after incubation in ISO media containing physiological Cl^−^. Results are expressed as nanomole/liter of Cl^−^ per microgram protein per unit of time. Each dot represents a single independent determination (*n*=6–9, *P<0.05). (D) Effect of chronic BTD pre-treatment on glucose-induced insulin secretion. MIN6 cells were cultured 16 h in media containing 5.5 mM glucose without (Control) or with 10 μM BTD. Then, BTD-treated MIN6 cells were divided into two groups: one was further incubated 2 h in KRBH plus vehicle (open dots) and another in KRBH plus 10 μM BTD (shaded dots). Insulin secretion was related to total insulin content (insulin secretion/total insulin content) and then normalized to basal levels (*n*=5, *P<0.05).

To assess the functional impact of the previous observations, we first determined the ability of MIN6 to accumulate Cl^−^ in a BTD-sensitive fashion. To this end, cells were depleted of endogenous Cl^−^ and allowed to recover the anion to basal values as a function of time under physiological conditions (ISO) or in the presence of 10 μM BTD (ISO+BTD). As shown in Fig. 5A, MIN6 accumulate Cl^−^ following a typical first-order kinetic curve with an estimated constant rate *k*=0.124/min, a maximal half-life *T*_1/2_=5.6 min and a plateau *P*=3.96 nmol/μg. However, BTD negatively impacted those kinetic parameters (*k*=0.111/min, *T*_1/2_=6.2 min and *P*=2.58 nmol/μg) indicating a BTD-dependent component of Cl^−^ uptake in MIN6, thus confirming and extending previous results (Sandstrom 1990, Best 2005).

Subsequently, we tested Cl^−^ accumulation in MIN6 as a function of increasing BTD concentrations to determine the IC_50_ for the diuretic. As shown in Fig. 5B, the BTD-sensitive component of Cl^−^ accumulation exhibits an estimated IC_50_ of ∼7.9 μM, a value well within the ranges described for NKCC1 in mammalian systems (Russell 2000). Therefore, MIN6 were pre-incubated 16 h with vehicle or BTD (1–50 μM), depleted of endogenous Cl^−^, and the initial rate of Cl^−^ accumulation was determined as total Cl^−^ uploaded in 5 min. As shown in Fig. 5C, 10 μM BTD maximally and significantly increases the initial rate of Cl^−^ influx into MIN6 β-cells unmasking mechanisms of Cl^−^ uptake engaged after chronic BTD treatment.

Because Cl^−^ fluxes participate in the stimulus-secretion coupling (Best 2005) and 10 μM BTD acutely inhibits insulin release in rodent islets and β-cells (Sandstrom 1990, Best 2005, Alshahrani & Di Fulvio 2012), we tested the secretory response of MIN6 pre-treated 16 h with BTD (10 μM) in the presence or absence of BTD during the insulin secretion assay (2 h). As shown in Fig. 5D, the insulin response of pre-treated MIN6 is inhibited when BTD is present in the assay buffer, but not when absent. Taken together, these results suggest the existence of a functional interplay between NKCC1 and NKCC2 aimed at modulating [Cl^−^]_i_ in β-cells to preserve the insulin secretory response.

## Discussion

These results show that β-cells are endowed with homeostatic mechanisms triggered in response to haploinsufficient *Slc12a2* aimed at restoring the secretory response. Indeed, NKCC1^HE^ mice exhibit significantly lower basal glycemia than NKCC1^WT^ (Fig. 1A), not explained by any other measurable changes or half NKCC1 expression in tissues (Flagella *et al*. 1999). NKCC1^HE^ and NKCC1^WT^ mice showed comparable glucose responses to exogenous insulin (Fig. 1B) but increased acute insulin secretion (Fig. 1C) and significantly lower glycemia 15–30 min after glucose load (Fig. 1D), all suggestive of improved rather than a deteriorated secretory capacity in NKCC1^HE^. Interestingly, the *in vitro* insulin response of NKCC1^HE^ islets was comparable to that of NKCC1^WT^ (Fig. 1E) despite reduced NKCC1 expression (Fig. 3K). This is compatible with compensatory mechanisms triggered in response to decreased levels of NKCC1 to maintain normal secretory responses.

This apparent discrepancy between insulin responses *in vitro* and *in vivo* could be attributed to positive or negative stimuli potentially at play *in vivo*, when the whole animal is characterized. Further, plasma insulin levels at a particular time point reflect the balance between insulin rates of biosynthesis, secretion and degradation, whereas insulin released by islets or β-cells *in vitro* reflects the stimulus-secretion coupling, which is under the sole control of glucose. Some of the compensatory mechanisms triggered by hemi-expression or absence of NKCC1 appear to be BTD-dependent, i.e., related to NKCCs. As mentioned, a single dose of BTD increases basal blood glucose in NKCC1^HE^ mice and deteriorates its disposal (Fig. 2A and B), matching the concept that NKCC1, NKCC2 or both participate in insulin secretion. Interestingly, the extent to which BTD increased basal glycemia and reduced glucose disposal in NKCC1^HE^ were similar to that of NKCC1^WT^ (Fig. 2B) suggesting equivalent effects of the diuretic in these mice, regardless of the *Slc12a2* gene dose. This could be attributed to a higher specific NKCC1 activity in NKCC1^HE^ islets relative to NKCC1^WT^. Indeed, basal NKCC activity is similar in astrocytes of NKCC1^HE^ and NKCC1^WT^ mice in spite of reduced total NKCC protein levels in NKCC1^HE^ compared to NKCC1^WT^ cells (Lenart *et al*. 2004).

Alternatively, the fact that mouse β-cells express NKCC2 (Alshahrani *et al*. 2012) and that its immunoreactive expression increased in NKCC1^HE^ and NKCC1^KO^ insulin-containing β-cells relative to NKCC1^WT^ (Fig. 3) supports the hypothesis that NKCC1 down-regulation increases NKCC2 expression in β-cells. Furthermore, chronic treatment of MIN6 with BTD, which results in decreased NKCC1 expression/function in other cells (Haas & Sontheimer 2010), triggered a dose-dependent decrease in NKCC1 expression in β-cells, paralleled by increased NKCC2 protein levels (Fig. 4A and B). It is important to notice that the maximal effect of the diuretic on the expression levels of NKCC1 and NKCC2 in MIN6 occurred at concentrations near the IC_50_ of the drug, i.e., 10 μM (Fig. 4B), hence, lessening the participation of other potential targets of BTD, also expressed in MIN6 (Di Fulvio *et al*. 2014) for which the BTD IC_50_ is much higher (Reddy & Quinton 1999, Alvarez-Leefmans 2012). Another supporting layer of the link between NKCC2 and NKCC1 expression comes from our experiments in COS7 cells, which express abundant NKCC1 (Singh *et al*. 2015) and very low levels of NKCC2 proteins (Fig. 4E, F and G). Like in MIN6, chronic BTD treatment decreased NKCC1 and increased NKCC2 protein expression in COS7 (Fig. 4H, I and J) indicating that the NKCC1/NKCC2 expression relationship may be a general event in cells co-expressing these two carriers. Most notably, chronic treatment of COS7 cells with BTD resulted in apparent redistribution of NKCC2 from internal compartments to the plasma membrane that correlated with evident cell shrinkage (Fig. 4H, I and J). This could be related to the known ability of NKCC1 to transport ions and water (Hamann *et al*. 2010), a property not shared by NKCC2, which only transports ions (Zeuthen & Macaulay 2012). Our results in combination with the fact that NKCC1 and NKCC2 are not co-expressed at comparable levels in endocrine cells including insulin- (Alshahrani *et al*. 2012), oxytocin- and vasopressin-secreting ones (Hindmarch *et al*. 2006, Konopacka *et al*. 2015) are compatible with the concept that NKCC2 cannot fully ‘compensate’ for NKCC1 functional absence.

Recent reports are suggestive of a functional interrelationship between changes in [Cl^−^]_i_ and expression of Cl^−^ channels or transporters. For instance, reduced [Cl^−^]_i_ in NKCC1-expressing neurons achieved either by overexpressing the Cl^−^ extruder KCC2 (*Slc12a5*) or by chronically treating them with BTD modulate expression of GABA_A_ Cl^−^ channels (Succol *et al*. 2012). In addition, NKCC2 expression in neurons of the hypotalamo-neuro-hypophyseal system increases in response to chronic dehydration (Hindmarch *et al*. 2006, Konopacka *et al*. 2015), a condition that can be elicited by chronic use of BTD (Jackson 2006). Regardless the fact that neurons and β-cells share many similarities (Atouf *et al*. 1997), the mechanistic details involved in the regulation of NKCCs in β-cells and the potential relationship to changes in [Cl^−^]_i_ remain to be addressed. Nevertheless, the physiological relevance of NKCC1 relative to NKCC2 in fuel homeostasis is highlighted by the following: the molecular and functional decrease of NKCC1 triggers enhanced NKCC2 expression, but not the other way around (Fig. 4D), NKCC1 transcripts in β-cells are at least ten times more abundant than those of NKCC2 (Alshahrani *et al*. 2012), NKCC1 comprises most of the NKCC protein pool in MIN6, COS7 (Fig. 4A and C) or neurons containing or releasing vasopressin (Belenky *et al*. 2010, Konopacka *et al*. 2015), most if not all NKCC2 in β-cells (Alshahrani *et al*. 2012, Alshahrani & Di Fulvio 2012), neuroendocrine (Konopacka *et al*. 2015) and COS7 cells (Fig. 4E, F and G) appears located to intracellular compartments, and acute inhibition of NKCCs with BTD rapidly reduced Cl^−^ uptake in MIN6 cells with an IC_50_ similar to that reported for NKCC1 (Fig. 5A and B).

While we recognize the limitations in identifying the underlying mechanism involved in BTD-sensitive Cl^−^ fluxes, chronic changes in the NKCC1/NKCC2 functional ratio due to reduced NKCC1 and augmented NKCC2 in β-cells may preserve [Cl^−^]_i_ in at least two ways: decreased cell water volume due to NKCC1 deficit and a normal or increased rate of Cl^−^ uptake due to NKCC2 up-regulation. This second point is supported by our results indicating increased initial rates of Cl^−^ uptake into MIN6 pre-treated 16 h with BTD (Fig. 5C). This correlates with ∼70% reduction in NKCC1 protein levels and a parallel increase of NKCC2 expression in MIN6 (Fig. 4A and B). Therefore, increased Cl^−^ uptake in MIN6 could be attributed, at least in part, to increased NKCC2 expression. Indeed, NKCC2 exhibits higher basal activity than NKCC1 (Hannemann & Flatman 2011). Irrespective of the potential mechanisms involved, the steady-state total Cl^−^ content in MIN6 pre-treated with BTD was not statistically significant (data not shown), suggesting that the functional consequence of increased NKCC2 expression due to decreased NKCC1 is the restoration of [Cl^−^]_i_. However, it is important to keep in mind that our experiments cannot discard the regulation of additional Cl^−^ loaders or extruders in response to chronic BTD treatment. At any rate, β-cells acutely treated with BTD have a blunted secretory response attributed to the rapid dissipation of the Cl^−^ gradient maintained by NKCC1 (Best 2005), explaining the intermittent hyperglycemia present in patients treated with diuretics (Jackson 2006). Although our results suggest that β-cells chronically pre-treated with BTD exhibit normal insulin secretory responses due to conserved [Cl^−^]_i_, 16 h pre-treatment with BTD blocked insulin release at all glucose concentrations tested only when the diuretic was present during the secretion assay but not when BTD was omitted (Fig. 5D). These results point toward a BTD-sensitive mechanism at play in the modulation of secretion in chronically treated MIN6, and our observations are in agreement with the notion that NKCC2 plays a minor role in insulin secretion, unless NKCC1 is reduced or eliminated. Taken together, our results unmask a functional interplay of two Cl^−^ loaders, i.e., NKCC1 and NKCC2, aimed at preserving [Cl^−^]_i_ and the insulin secretory response in pancreatic β-cells.

## Author contribution statement

All authors participated in the experimental design, data analysis and interpretation and revised the manuscript for intellectual content. M M A carried out protein expression analyses, Cl^−^ uptake and insulin secretion studies in MIN6. S A and M D F performed dynamic tests of glucose homeostasis in mice, genotyping, islet studies and COS7 immunofluorescence microscopy. S K and E D-J performed tissue processing, antibody characterization and immunofluorescence microscopy experiments in tissues. M M A and M D F produced, maintained and characterized stable cells. L A-B critically read the manuscript and participated in very fruitful discussions. M D F conceived the studies and wrote the manuscript.
